# Integrating Acceptance and Commitment Therapy with intervention mapping to enhance self-care in type 2 diabetes: a conceptual framework

**DOI:** 10.3389/fpsyg.2026.1822037

**Published:** 2026-06-15

**Authors:** Paula Helena Gomes de Moraes Ruiz, Luziane de Fátima Kirchner, Elisa Kern de Castro

**Affiliations:** 1 Dom Bosco Catholic University (UCDB), Campo Grande, MS, Brazil; 2 Egas Moniz Center for Interdisciplinary Research (CiiEM), Almada, Portugal; 3 Egas Moniz School of Health & Science, Almada, Portugal

**Keywords:** Acceptance and Commitment Therapy, chronic disease management, health behavior change, intervention mapping, psychological flexibility, self-care, type 2 diabetes

## Abstract

Self-care in type 2 diabetes mellitus (T2DM) remains suboptimal despite evidence for the effectiveness of Acceptance and Commitment Therapy (ACT) in chronic illness management. This conceptual paper integrates ACT-based psychological flexibility processes with Steps 1-4 of the Intervention Mapping framework to develop a brief, replicable group intervention for primary care. Acceptance, cognitive defusion, and values clarification are specified as modifiable determinants of barriers in self-monitoring, diet, exercise, and medication adherence, organized into performance-determinant matrices to guide method selection. Theory-based methods are operationalized through culturally adaptable metaphors and experiential exercises. This framework addresses methodological gaps in translating ACT to diabetes care, aligns with biopsychosocial models in health psychology, and offers a testable foundation for future randomized trials on multicultural efficacy.

## 1 Introduction

Type 2 diabetes mellitus (T2DM) accounts for approximately 90% of global diabetes cases and ranks among the leading causes of morbidity and mortality from non-communicable chronic diseases (World Health Organization, 2019; International Diabetes Federation, 2025). Its pathophysiology is closely linked to genetic and environmental factors such as population aging, physical inactivity, and obesity, which pose additional challenges to preventing chronic complications (Riddle et al., 2021; Mohr et al., 2022; Genitsaridi et al., 2025). Recent projections indicate that global diabetes prevalence—currently affecting hundreds of millions—will continue rising over the coming decades (World Health Organization, 2019; International Diabetes Federation, 2025), further straining healthcare systems and compromising quality of life for individuals with T2DM. In this context, self-care emerges as the cornerstone of management, encompassing glycemic monitoring, medication adherence, dietary adjustments, and regular physical activity, as recommended by international guidelines.

Empirical evidence indicates that ACT improves self-care in patients with type 2 diabetes (Abdollahi et al., 2020; Wang et al., 2024; Ahmadi et al., 2025; de Moraes Ruiz et al., 2025). However, translating these findings into clinical practice faces significant methodological gaps. The available studies show heterogeneity in intervention formats, with insufficient descriptions of the operationalization of ACT processes, which compromises replicability and the identification of the change mechanisms involved (Sakamoto et al., 2021; Wang et al., 2024; Batool and Mubashir, 2025; de Moraes Ruiz et al., 2025). Furthermore, even when the results are promising, translating these findings into clinical practice remains hampered by the absence of strategies that clearly guide the implementation of ACT in real-world healthcare settings.

In this context, the Intervention Model (IM) presents itself as a useful methodological framework to support the operationalization of ACT in single-session interventions aimed at type 2 diabetes (T2D), which become particularly relevant in primary care. Unlike approaches focused solely on describing therapeutic content, the IM provides a systematic protocol for connecting theory, behavioral determinants, change objectives, and intervention techniques, favoring greater rigor in the construction and implementation of programs (Kok et al., 2016; Bartholomew et al., 2016; Fernandez et al., 2019; Herbert et al., 2025). In particular, the initial steps of the IM allow for the identification of critical determinants of self-care in T2D—such as perceived barriers, experiential avoidance, self-efficacy, and treatment adherence—and their translation into change objectives operationalized by ACT processes.

This conceptual paper applies IM Steps 1–4 systematically to develop an intervention that operationalizes core ACT processes as behavioral change determinants for T2DM self-care in adults. Specific objectives include:

1. Identify ACT processes (acceptance, cognitive defusion, values) as modifiable psychological determinants of T2DM self-care barriers (IM Steps 1–2);
2. Build a change objectives matrix crossing self-care behaviors with ACT processes (IM Step 2);
3. Select and operationalize ACT theoretical methods into replicable practical applications for brief primary care interventions (IM Step 3);
4. Integrate practical applications into a structured single-session group program (IM Step 4).

Based on these objectives, the manuscript is guided by the following research question: how can ACT processes be systematically mapped and operationalized within the IM protocol to optimize self-care in individuals with type 2 diabetes? Two aspects are central to situating this proposal. First, the manuscript presents a conceptual and planning structure restricted to the first four stages of IM, which constitute the theoretical and methodological basis necessary for the development and refinement of the protocol. Feasibility, implementation, and effectiveness evaluation studies, corresponding to Stages 5 and 6 of IM, are beyond the scope of this article and are indicated as future research agendas. Second, the single-session protocol was intentionally formulated based on criteria of feasibility, applicability, and potential scalability in primary care, being directed at professionals with expertise in ACT and conceived in a brief and structured format, compatible with the time and resource constraints of health services. Furthermore, the protocol integrates an ongoing research project in Brazil (Kirchner et al., 2025), in which initial implementation and evaluation studies are being carried out.

This article comprises four main sections, plus this introduction. First, we outline T2DM's epidemiological and psychosocial challenges, highlighting limitations of traditional educational approaches to sustained self-care. Next, we present ACT's conceptual foundations, detailing Hexaflex processes and their interface with chronic disease management. The third section examines IM as a systematic, evidence-based planning tool. Finally, the analytical section details the integration of ACT-IM's theoretical-methodological framework, culminating in the construction of a change objectives matrix and primary care-oriented practical applications, followed by final considerations on model limitations and future research directions.

## 2 Type 2 diabetes mellitus: self-care challenges beyond knowledge

T2DM manifests as a chronic metabolic disorder with multifactorial etiology, primarily defined by macronutrient metabolism dysregulation. Pathophysiologically, the condition arises from complex interactions among peripheral insulin resistance, deficient insulin secretion by pancreatic beta cells, and excessive hepatic glucose production (Song et al., 2023; American Diabetes Association Professional Practice Committee, 2024). Epidemiologically, T2DM accounts for the vast majority of cases and constitutes a critical global public health challenge. The International Diabetes Federation (2025) reports the current prevalence at 589 million people, projected to reach 853 million by 2050 without effective control. This scenario proves especially severe in low- and middle-income countries, driven by nutritional transitions, rapid urbanization, and sedentary lifestyles that exacerbate modifiable risks such as elevated body mass index and poor dietary patterns (Mokaya et al., 2022; Van Den Bekerom et al., 2024).

Chronic hyperglycemia from T2DM, if unmitigated, serves as the central mechanism driving severe complications. Inadequate care leads to outcomes including retinopathy, nephropathy, neuropathy, cardiovascular disease, and foot complications that increase amputation risk (Chamine et al., 2022; Rietz et al., 2022; Schrader et al., 2022; American Diabetes Association Professional Practice Committee, 2024). Glycemic control thus plays a predominant role, as literature shows each 1% reduction in hemoglobin A1c (HbA1c) levels is associated with significant decreases in these morbidities and acute events like myocardial infarction (Almulhim et al., 2023; Butayeva et al., 2023).

However, T2DM complexity extends beyond biology, presenting as a systemic condition with psychosocial and economic implications. Disease management imposes a psychological burden marked by negative emotional responses to diagnosis and ongoing self-care demands (Bhat et al., 2020). Patients exhibit high rates of psychiatric comorbidities such as depression and anxiety, plus evidence that T2DM impairs cognitive flexibility (Maor et al., 2022; Motevalli et al., 2023). Emotional distress and cognitive deficits hinder adherence to therapeutic regimens, perpetuating glycemic dysregulation. Proper diabetes management requires continuous patient engagement and can sometimes be compromised by the interaction of factors such as emotional distress, manifested through chronic stress, anxiety, or depression associated with the disease, which acts as a significant barrier interfering with self-care routines (Ahmadi et al., 2025; Ho et al., 2024; Wang et al., 2024). To cope with this burden, individuals may merge with cognitions of incapacity and resort to experiential avoidance in an attempt to suppress the emotional discomfort associated with the disease and obtain short-term relief. This continuous cycle of avoidance diverts the patient's attention from the present moment and paralyzes effective action, preventing them from performing self-care actions based on their values (Gregg et al., 2007a). Consequently, this behavioral neglect motivated by these factors perpetuates glycemic dysregulation (Ahmadi et al., 2025; Ho et al., 2024; Wang et al., 2024), which hinders the reduction of glycated hemoglobin (HbA1c) levels and aggravates the morbidity of the chronic condition.

Effective T2DM management thus requires rigorous self-care and sustainable behavioral changes. Current clinical guidelines (International Diabetes Federation, 2025) recommend multimodal interventions including pharmacotherapy, glycemic monitoring, healthy eating, and physical activity. Yet the disease's often asymptomatic nature undermines patients' gravity perception, creating a paradox where control benefits emerge long-term while treatment demands prove immediate and burdensome (Porth et al., 2024). Given this complexity, strengthening self-care capacity emerges as the central element ensuring favorable clinical outcomes and quality of life.

T2DM self-care extends beyond passive medication adherence. Under Orem's Self-Care Theory, it requires active, intentional performance to maintain life and wellbeing (Fereidooni et al., 2024; Esmaeilzadeh and Inuwa, 2025). In clinical practice, T2DM self-care behaviors encompass integrated management of multiple behavioral classes, including continuous glycemic monitoring, medication adherence, dietary adjustments, and regular physical activity (International Diabetes Federation, 2025).

However, literature shows that clinical guidelines and knowledge of recommendations do not necessarily translate to sustained metabolic control. Although education and support standards exist (Butayeva et al., 2023), practical implementation faces low utilization, limiting population health impact (World Health Organization, 2024). Traditional information transmission approaches often fail to ensure long-term self-care, as most patients maintain poor adherence under standard care regimens (Aminuddin et al., 2021; Hyvert et al., 2023; Parmar et al., 2025).

Epidemiological data confirm unsatisfactory self-care rates, leaving many patients below recommended glycemic targets (HbA1c <7%), which increases morbidity, mortality, and healthcare costs (Genitsaridi et al., 2025). This knowing-doing gap exposes limitations of purely educational approaches (Aminuddin et al., 2021; Hyvert et al., 2023; Parmar et al., 2025). Traditional interventions, focused on prescriptive norms, overlook that behavioral inaction stems not only from information scarcity (Batool and Mubashir, 2025; Zandi et al., 2023) but also from psychological determinants like experiential avoidance (Ho et al., 2024) and the disease's short-term asymptomatic nature (Porth et al., 2024), which favors immediate relief over long-term self-care.

In this context, experiential avoidance and cognitive fusion emerge as key psychological barriers to self-care. Patients often avoid self-care behaviors (e.g., skipping glycemic testing) to suppress chronic condition-related emotional discomfort or fuse with inability cognitions (e.g., “diabetes is uncontrollable”), paralyzing effective action (Gregg et al., 2007a; Ho et al., 2024). Conventional health education rarely addresses these barriers, perpetuating metabolic dysregulation cycles.

Given these informational model limitations, Acceptance and Commitment Therapy (ACT) offers an alternative. By promoting psychological flexibility (PF), ACT equips patients to accept treatment-inherent discomfort without abandoning self-care, redirecting behavior toward personal values rather than symptom reduction alone (Ho et al., 2024). Although empirical evidence confirms ACT improves T2DM self-care (Abdollahi et al., 2020; Wang et al., 2024; Ahmadi et al., 2025; de Moraes Ruiz et al., 2025), clinical translation faces methodological gaps. Current literature reveals intervention format heterogeneity and lacks detailed ACT process operationalization, hindering replicability and change mechanism understanding (Sakamoto et al., 2021; Wang et al., 2024; Batool and Mubashir, 2025; de Moraes Ruiz et al., 2025).

To address this systematization gap, this conceptual paper proposes Intervention Mapping (IM). IM provides an evidence-based planning structure (Kok et al., 2016; Bartholomew et al., 2016; Fernandez et al., 2019; Herbert et al., 2025) that systematically links theory (ACT) to practice, step by step. This integration bridges the gap between ACT's theoretical efficacy and the need to identify elements for replicable, contextually applicable T2DM care protocols.

## 3 Acceptance and Commitment Therapy and its relation to diabetes

Psychological flexibility (PF) refers to the ability to fully contact the present moment consciously and persist in or change behavior based on context to serve valued ends (Hayes et al., 2021; Hayes and Hofmann, 2021; Hayes and King, 2024). Promoting PF, ACT's central focus, shows clinical utility by fostering personal growth and alleviating human suffering through contextual understanding of individual interactions with private and external environments (Luoma et al., 2022; Hofman et al., 2023). ACT conceptualizes psychological health and PF development through six interconnected processes, the “Hexaflex,” essential for T2DM self-care:

1. Acceptance: Patients actively experience private events (emotions, physical sensations) without attempts to alter or suppress them (Hayes et al., 2021; Luoma et al., 2022; Hofman et al., 2023). In T2DM, this means experiencing hypoglycemia anxiety or cravings for restricted foods without impulsive reactions or avoiding necessary monitoring.
2. Cognitive Defusion: Patients observe thoughts as transient mental events rather than literal truths (Hayes et al., 2021; Luoma et al., 2022; Hofman et al., 2023). This counters cognitions like “I am a sick diabetic” or “I will never control my diet,” reducing their impact and literalness.
3. Contact with the Present Moment: Patients maintain flexible, focused, non-judgmental attention to current experience (Hayes et al., 2021; Luoma et al., 2022; Hofman et al., 2023). This proves essential for detecting subtle bodily signals (hunger, satiety, glycemic symptoms) and interrupting autopilot unhealthy habits.
4. Self-as-Context: Patients perceive an observing self distinct from experiences (disease, thoughts) (Hayes et al., 2021; Luoma et al., 2022; Hofman et al., 2023). This allows viewing T2DM as something patients have, not what they are, preserving identity and self-esteem beyond pathology.
5. Values: Patients clarify what matters most to them (Hayes et al., 2021; Luoma et al., 2022; Hofman et al., 2023). Values provide sustainable self-care motivation, transforming medical obligation into life service (e.g., self-care to witness grandchildren's growth).
6. Committed Action: Patients build effective, concrete behavior patterns guided by identified values (Hayes et al., 2021; Luoma et al., 2022; Hofman et al., 2023). This involves goal setting and action persistence despite aversive private events.

T2DM clinical scenarios frequently manifest PF absence as psychological inflexibility (Batool and Mubashir, 2025). Patients fuse with aversive internal experiences like “nothing I can do changes my condition” and engage in experiential avoidance for short-term relief (Ho et al., 2024). This avoidance cycle often leads to glycemic control neglect and poor treatment adherence, perpetuating suffering (Motevalli et al., 2023). Defusion and acceptance processes, conversely, promote PF, enabling present-moment attention redirection and effective values-based self-care actions (Gregg et al., 2007a; Abdollahi et al., 2020; Wang et al., 2024).

Diabetes diagnosis demands substantial lifestyle changes and self-care behaviors to prevent complications. Emotional management fundamentally shapes self-care, as disease-averse events like dietary restrictions or retinopathy risk frequently elicit fear responses (Song et al., 2023). Ineffective control measures driven by fear, aimed at discomfort suppression, establish negative reinforcement cycles that exacerbate emotional suffering and deteriorate metabolic control (Motevalli et al., 2023). Cognitive fusion and experiential avoidance thus function as self-care barriers, diverting patient attention to past rumination or anticipatory anxiety (Wang et al., 2025).

This present-moment distancing impairs capacity to act on controllable variables, reducing daily disease management efficacy. Fusion with aversive private events disconnects individuals from immediate contingencies, frequently resulting in essential health behavior abandonment (Abdollahi et al., 2020). ACT-based interventions prove effective precisely by promoting psychological flexibility (Hayes and King, 2024; Ahmadi et al., 2025; Moravejosharieh and Dasht Bozorgi, 2025). This skill development maintains conscious, open “here and now” experience, reducing experiential avoidance's paralyzing influence (Kiliç et al., 2023). ACT equips patients—through core processes like acceptance and committed action—to persist in self-care behaviors like dietary adherence and glycemic monitoring despite emotional discomfort or contextual barriers (Ngan et al., 2023; Moravejosharieh and Dasht Bozorgi, 2025).

Systematic reviews indicate ACT superiority over control groups and comparability or superiority to other approaches like CBT in improving T2DM self-care outcomes (de Moraes Ruiz et al., 2025). Seminal experimental studies corroborate that adding ACT to educational training significantly increased coping strategy probability and improved glycemic control in vulnerable populations (Gregg et al., 2007a). Convergent results appear across cultural contexts and intervention modalities, including in-person formats (Wang et al., 2024) and mobile technology-mediated interventions (Kiliç et al., 2023), confirming ACT's positive impact on hemoglobin A1c reduction and self-care promotion. Research with younger populations further indicates experiential avoidance management as a key self-care mechanism, suggesting psychological process transversality (Alho et al., 2022).

Clinical translation nevertheless faces methodological barriers. Literature reveals substantial application format heterogeneity—from single sessions to group therapies—often lacking detailed therapeutic process operationalization descriptions (de Moraes Ruiz et al., 2025). Many interventions fail to specify ACT process articulation with T2DM-specific practical challenges, creating replicability and precise change mechanism comprehension problems. This systematization gap highlights the need for protocols that not only adapt ACT but integrate it with evidence-based planning structures. IM protocol emerges as the ideal methodological tool, systematically translating ACT theoretical methods into replicable practical applications.

## 4 Intervention mapping

Intervention Mapping (IM) comprises a six-step planning protocol that links theory, behavioral determinants, and intervention techniques. The six steps and related tasks include (Kok et al., 2016; Bartholomew et al., 2016; Fernandez et al., 2019; Herbert et al., 2025):

- **Step 1**: Conduct a needs assessment or problem analysis to identify what needs change and for whom.
- **Step 2**: Create objective matrices combining behaviors with behavioral determinants to target specific beliefs.
- **Step 3**: Select theory-based intervention methods matching identified determinants and translate them into practical applications meeting efficacy parameters. This involves key program design decisions, including components, scope, sequence, and behavior change methods.
- **Step 4**: Integrate practical applications into an organized program.
- **Step 5**: Plan program adoption, implementation, and sustainability in real contexts, identifying users and supporters and addressing their needs.
- **Step 6**: Develop an evaluation plan for effect and process evaluations measuring program efficacy.

IM's keywords—planning, research, theory—provide vocabulary for program planning, activity procedures, and technical assistance to identify theoretical determinants and match them with appropriate change methods (Kok et al., 2016; Herbert et al., 2025). Intervention mapping guides effective health promotion material development across diverse contexts and populations (Mirzaei-Alavijeh et al., 2021; Halliday et al., 2021; Van Agteren et al., 2021; Mozafarinia et al., 2024), demonstrating efficacy in determinant identification and disease prevention adherence.

Successful intervention fundamentally depends on precise identification of behavioral and environmental determinants, core processes in the protocol's first two steps. Intervention efficacy directly hinges on this diagnostic phase, as recent study analyses illustrate (Mirzaei-Alavijeh et al., 2021). In T2DM contexts, experimental evidence shows structured interventions targeting specific theoretical determinants—perceived barriers, outcome expectations, self-efficacy—yield significant improvements in medication and dietary adherence plus HbA1c reduction (Ranjbaran et al., 2022).

Step 2 operationalizes these findings through “change objective matrices.” This tool crosses performance objectives (what individuals do) with modifiable determinants (why they do it), creating logical behavior change structures (Van Agteren et al., 2021). Literature confirms this approach's utility through effective alignment of diet and physical activity objectives with self-regulation determinants in diabetes patients (Ranjbaran et al., 2022; Song et al., 2023).

Once objectives define, researchers must distinguish theoretical methods from practical applications. Translation losses frequently occur when methods lack adequate implementation, violating efficacy parameters. IM addresses this gap in Step 3 by requiring concrete theory operationalization. For example, goal setting alone shows limited effects unless systematic (Mozafarinia et al., 2024); theoretical integrity requires faithful method translation, such as modeling videos respecting pedagogical parameters (Halliday et al., 2021).

Intervention internal and external validity ultimately depends on implementation fidelity addressed in IM's final steps. Adoption and evaluation planning ensure delivered interventions match planned ones, as exemplified in ACT-based mHealth intervention development (Nes et al., 2018). Qualitative analysis confirmed high clinical protocol adherence using IM principles to structure provider training and delivery monitoring, validating study integrity. IM thus constitutes an essential research tool, ensuring interventions prove both theoretically grounded and methodologically executable in health contexts.

## 5 Integrating intervention mapping and ACT for type 2 diabetes interventions

Literature firmly establishes ACT's relevance for T2DM self-care and IM's utility as a planning tool, yet their intersection remains underexplored. Key issues emerge: ACT-based T2DM interventions, though clinically relevant, lack detailed systematization that hinders replication and fidelity (de Moraes Ruiz et al., 2025); meanwhile, IM-developed programs, despite structural rigor, often limit themselves to traditional cognitive models (Halliday et al., 2021; Mirzaei-Alavijeh et al., 2021), neglecting psychological flexibility processes crucial for sustained self-care (Nes et al., 2018).

This conceptual paper proposes an integration where ACT's core processes operationalize as theoretical change determinants within the IM protocol. Specifically in Steps 1 and 2—grounded in prior literature analysis of problems and determinants (Nes et al., 2018; Herbert et al., 2025)—this proposal reinterprets change mechanisms.

Traditionally, IM's Change Objectives Matrix (Step 2) targets thought content modification (e.g., boosting self-efficacy or altering attitudes). This integrative proposal expands and functionally replaces classic determinants with PF processes. Objectives shift from controlling symptoms or thoughts to altering patients' relationship with private events (function), prioritizing Acceptance, Cognitive Defusion, and Committed Action.

The Change Objectives Matrix construction (Table 1) rests on literature consensus regarding PF's centrality as a transdiagnostic change mechanism (Hayes and King, 2024; Moravejosharieh and Dasht Bozorgi, 2025). Unlike traditional cognitive approaches focusing on thought content restructuring, ACT alters patients' function and relationship with private events (Zhang et al., 2018; Zandi et al., 2023; Hayes and King, 2024). This paradigm shift provides the theoretical foundation justifying classic determinant replacement with ACT processes in this intervention proposal.

**Table 1:** Change objectives matrix: crossing self-care behaviors with ACT processes.

Performance objective (what will patients do?)	Determinant: acceptance	Determinant: cognitive defusion	Determinant: values
Perform prescribed glycemic monitoring.	Experience aversive sensations (lancet pain, result anxiety) as concomitant to action without suppressing or avoiding the test.	Distinguish catastrophic thoughts (e.g., “the result will be terrible” or “I don't want to know”) from present reality, treating them as verbal events, not action commands.	Patients articulate how monitoring serves their values (e.g., “care for me to be a present grandfather”).Artigo-1-Paula-27-01.docx?
Follow dietary plan in challenging social situations.	Accept deprivation sensations or intense cravings in social contexts while maintaining pre-planned food choices despite discomfort.	Observe permissive rationalizations (e.g., “just today won't hurt”) without fusing with them or using them to justify diet breaks.	Patients choose nutritious foods not as external imposition but as self-care and body respect aligned with vitality values and commitment.Artigo-1-Paula-27-01.docx?
Engage in regular physical activity.	Embrace inherent physical discomfort from exercise (sweat, mild fatigue) as self-care evidence without interrupting activity.	Notice fatigue or demotivation evaluations (e.g., “I'm too tired”) as transient mental events, acting independently of thought content.	Patients link walking to long-term physical independence and mobility maintenance.Artigo-1-Paula-27-01.docx?
Adhere to medication (insulin or oral).	I am willing feel anticipatory anxiety or injection aversion while administering the dose in the presence of these emotions.	Use the deliteralization process for stigmatizing beliefs (e.g., “insulin use signals personal failure”), recognizing them as learned judgments not absolute truths.	Patients view medication as a tool enabling life projects and valued actions continuity.Artigo-1-Paula-27-01.docx?

Based on Zhang et al. (2018), Abdollahi et al. (2020), Ngan et al. (2023), Moravejosharieh and Dasht Bozorgi (2025), Lipsett and Berkman (2025), and Ahmadi et al. (2025).

This structure's first pillar addresses experiential avoidance. In T2DM, patients frequently avoid self-care behaviors (e.g., glycemic monitoring) because these evoke aversive feelings of guilt, fear, or physical discomfort. Abdollahi et al. (2020) explain that internal experience suppression efforts consume substantial cognitive resources, depleting mental energy needed for complex disease management. Consequently, the intervention targets acceptance promotion (Table 1, Column 2). Moravejosharieh and Dasht Bozorgi, 2025 statistical evidence supports this choice, showing that avoidance strategy reduction frees patients to focus on problem-solving. The goal thus becomes enabling patients to perform glucose testing *in the presence of* fear, not eliminating fear.

A second pillar addresses cognitive fusion, where patients treat incapacity thoughts (e.g., “diabetes is my life sentence”) as literal truths, culminating in inaction (self-care absence). The intervention thus employs cognitive defusion (Table 1, Column 3). Ngan et al.'s (2023) qualitative studies indicate that learning to notice thoughts without being controlled by them reduces emotional reactivity. This enables health behaviors to persist regardless of daily private events, ensuring treatment continuity even during aversive T2DM thoughts.

Finally, self-care sustainability requires values reorientation. Literature suggests that fear-based or medical obedience-based self-care proves fragile long-term (Zhang et al., 2018; Lipsett and Berkman, 2025; Ahmadi et al., 2025). In contrast, ACT introduces values-based mechanisms (Table 1, Column 4). Abdollahi et al. (2020) describe how clarifying patient-important domains (e.g., vitality to witness grandchildren's growth) transforms self-care from external imposition to a meaningful life step. Ahmadi et al. (2025) suggest these intrinsic values anchoring differentiates ACT results' resilience compared to isolated behavioral activation techniques.

In summary, this process integration aims not merely at symptom reduction but at strengthening adaptive coping repertoires. This PF- and values-based approach provides the necessary theoretical foundation for operationalizing detailed change objectives below (Abdollahi et al., 2020; Wang et al., 2024).

Thus, Table 1 presents this structure, where essential performance objectives for T2DM self-care and control (rows)—such as glycemic monitoring and medication adherence—cross with ACT therapeutic processes (columns). The result yields learning objectives that target not discomfort elimination but individual capacity to act toward personal values *with* discomfort present, breaking inaction cycles driven by fusion and avoidance.

Continuing IM's sequential logic, Step 3 involves selecting theoretical methods capable of influencing identified determinants (ACT processes) and translating them into clinically applicable intervention strategies. This protocol's construction draws on PF literature (Hayes et al., 2021; Luoma et al., 2022; Hofman et al., 2023), integrating Kok et al. (2016) feasibility parameters that emphasize technique alignment with healthcare system resources.

Selection followed this flow: (1) isolate the core ACT process needed to overcome psychological barriers (e.g., Acceptance to counter Experiential Avoidance); (2) define the change mechanism (e.g., recontextualization or distancing); and (3) operationalize into metaphors or experiential exercises adapted to T2DM patient reality. Therapeutic metaphors serve as the primary vehicle due to their efficacy in altering patient-suffering relationships without direct confrontation or logical argumentation—approaches that often fail to sustain change (Hayes et al., 2021; Luoma et al., 2022).

Table 2 details this strategic alignment. To operationalize acceptance, we selected the “Bicycle Metaphor.” This physically illustrates diabetes control paradox: just as excessive handlebar rigidity causes falls, suppressing hypoglycemia or disease fear generates metabolic dysregulation; the intervention teaches patients to “lean in” (accept) to maintain balance and direction.

**Table 2:** Operationalization of ACT methods within the IM protocol.

ACT process (target determinant)	Theoretical method (change mechanism)	Practical application (specific technique/metaphor)	Description in diabetes context
Acceptance (target: experiential avoidance)	Recontextualization via metaphor: shift aversive private events function from “danger signals” to “part of process,” promoting behavioral willingness.	Bicycle metaphor (riding a bicycle)	Illustrates T2DM self-care control paradox: excessive rigidity (fall fear/avoidance) creates instability, while “leaning forward” (risk/discomfort acceptance) enables balance and movement. Patients learn glycemic control (pedaling) requires accepting inherent disease uncertainty.
Committed action (target: lack of engagement/inaction)	Public commitment and behavioral activation: use social contract and physical posture to boost intention and facilitate response initiation.	“Stand up and commit” exercise	Physical/symbolic exercise where group members stand and verbally declare before the group their diabetes identity aspirations and specific self-care actions (goals). Physical standing act increases commitment, breaking passivity by linking intention to immediate bodily action.
Values/creative hopelessness (target: extrinsic motivation/ineffective control)	Functionality analysis: confront emotional control strategy inefficacy, redirecting to values-based action/choice.	“You have a choice” discussion	Structured dialogue distinguishing biological control (possible/necessary) from emotional control (impossible/exhausting). Demonstrating “fighting T2DM” emotionally equals trying to “unsee” something; therapist redirects patients to their sole controllable variable: present health behavior choices.
Cognitive defusion (target: fusion with disease thoughts)	Event discrimination (internal vs. external): teach distancing and private event observation.	“Trash on the ground” discussion	Analogy differentiates external problems (ground trash, sweepable) from internal problems (diabetes thoughts, un-sweepable). Technique teaches patients treat catastrophic thoughts not as sweepable trash but background noise not impeding self-care action.

Intervention based on Gregg et al. (2007a,b).

To counter cognitive fusion, the private events distancing method translates into the “Trash on the Ground” analogy. This functionally differentiates physical world problems (removable, like sweeping trash) from internal events (thoughts that persist or intensify under suppression attempts), promoting defusion from ineffective mental control strategies.

Finally, to overcome self-care inertia through committed action, we mapped the “Stand Up and Commit” experiential exercise. Asking participants to physically stand before the group to declare a health goal transcends cognitive intention, leveraging body posture and public commitment to boost behavioral salience and execution probability. Notably, all applications suit brief intervention formats (single or group sessions of 90–120 min) and align with seminal Gregg et al. (2007a,b) research, ensuring primary care implementation feasibility.

Regarding IM Step 4, researchers integrate selected practical applications into an organized program, defining scope, sequence, and materials. Intervention format decisions followed primary care feasibility parameters, where time and human resource scarcity pose constant barriers (Ho et al., 2024).

Thus, we defined a group format intervention scope that optimizes professional resources (Ho et al., 2024; Wang et al., 2024) while fostering peer learning and social support (Ho et al., 2024; Zu et al., 2024). The single-session structure, estimated at 90–120 min, follows Hexaflex logic: beginning with creative hopelessness (validating suffering and prior control failure), progressing to values construction and committed action.

This configuration aligns with literature showing brief, structured group interventions achieve good acceptability, higher adherence, and lower dropout rates compared to extensive models, particularly in public health and chronic condition management (Strosahl et al., 2012; Dochat et al., 2021; Stefanescu et al., 2024). Evidence indicates concise protocols anchored in transdiagnostic principles like ACT core processes maintain clinical efficacy while enhancing implementation feasibility in time- and resource-constrained services (Strosahl et al., 2012). Moreover, standardized intervention flow and experiential metaphors/exercises promote model fidelity, reduce high clinical expertise dependence, empower multiprofessional team training, and support protocol scalability without compromising behavioral and psychosocial outcomes.

IM Steps 5 and 6 address implementation planning and program evaluation systematization, respectively. Since empirical execution of these phases exceeds this article's analytical scope, subsequent methods and results will appear in dedicated literature. However, this protocol's implementation design accounts for global mental health access disparities, aligning with contemporary ACT dissemination trends in low- and middle-income countries (Lim et al., 2024).

For future efficacy evaluation (IM Step 6), the proposed design extends beyond conventional biological markers. While HbA1c reduction remains the primary clinical outcome (Mohr et al., 2022; Genitsaridi et al., 2025), ACT's theoretical foundation requires psychological mediator measurement. Thus, the evaluation plan must assess T2DM-specific psychological flexibility, which frequently mediates behavioral intervention-metabolic outcome relationships. Finally, researchers recommend clinical trials evaluating not only statistical significance but also social validity and patient perspectives on proposed metaphor acceptability.

## 6 Discussion

This conceptual paper achieved its primary objective by developing a theoretical-methodological framework that integrates Acceptance and Commitment Therapy (ACT) with the Intervention Mapping (IM) protocol for type 2 diabetes mellitus (T2DM) management. The research trajectory reveals that purely educational approach limitations stem not from information scarcity but from neglecting functional psychological determinants—specifically experiential avoidance and cognitive fusion.

This work contributes by systematizing psychological flexibility (PF) as a change mechanism within IM's logical matrix. By replacing traditional cognitive determinants with Hexaflex processes in the Change Objectives Matrix, the proposed protocol shifts intervention focus from symptomatic or cognitive control to altering patients' functional relationship with private events. Moreover, operationalizing theoretical constructs into tangible practical applications—like translating acceptance and committed action into metaphors and experiential exercises—addresses the literature gap regarding ACT-based intervention replicability, demonstrating theoretical integrity maintenance while meeting public health standardization criteria.

However, researchers must recognize this research's inherent scope limitations. Methodologically, the paper focused on intervention planning (IM Steps 1–4), excluding empirical execution and clinical outcome evaluation, preventing proposed brief group format efficacy verification at this stage. Additionally, the model's initial theoretical homogeneity warrants consideration. The protocol excludes severe psychiatric comorbidity intervening variables (mood or personality disorders), which frequently co-occur with T2DM and may require therapeutic dosage adaptations or metaphor flexibility adjustments.

A critical point concerns protocol transcultural and clinical generalizability. Since ACT's theoretical foundation and metaphors predominantly derive from Western matrices, techniques like cognitive defusion—dependent on linguistic and symbolic resonance—lack validation in low health literacy populations or extreme social vulnerability contexts (Hayes et al., 2021). Evidence from cultural adaptations in T2DM interventions for Asian Americans shows “cultural tailoring”—including native language materials, ethnically compatible Community Health Workers (CHWs), and local foods/exercises (e.g., tai chi)—improves retention and glycemic outcomes but requires ethnic subgroup phenotyping due to heterogeneity (Lim et al., 2024). Similarly, meta-analyses confirm ACT efficacy for T2DM self-care in Asian contexts (e.g., China, Iran), though format and population heterogeneity suggests local validation needs for processes like acceptance and values (Wang et al., 2024). In these scenarios, systemic and structural self-care barriers may exceed individual psychological determinants, requiring caution in direct model transposition without cultural adaptations as proposed in IM Step 1 for local needs. To mitigate this, researchers recommend integrating T2DM global disparities—higher prevalence in low- and middle-income countries (LMICs), projecting 853 million cases by 2050 (International Diabetes Federation, 2025)—into IM Step 1 through local needs assessments incorporating socioeconomic and cultural barriers. Future cultural adaptation studies could refine ACT metaphors and processes for non-Western contexts, ensuring Change Objectives Matrix functionality across diverse populations (Lim et al., 2024; Wang et al., 2024).

### 6.1 Future research directions

This theoretical construction invites continued scientific investigation through specific designs. Future studies should prioritize:

- **Feasibility and pilot studies** implementing the developed protocol in primary care to assess patient acceptability and organizational viability.
- **Multicultural randomized controlled trials (RCTs)** testing protocol efficacy against usual care, measuring self-care, PF, and HbA1c outcomes across diverse populations to validate Change Objectives Matrix functionality in varied sociocultural contexts. RCTs should adopt biopsychosocial designs measuring PF, self-care, and HbA1c in diverse populations (e.g., LMICs), aligning with *Frontiers in Psychology*'s scope for health psychology advances emphasizing biopsychosocial perspectives and population diversity.
- **Cultural adaptation and viability studies** refining practical applications through qualitative research to ensure metaphors resonate with local values, plus evaluating non-specialist implementation, and organizational feasibility.
- **Subgroup and moderator analyses** investigating intervention efficacy across T2DM patients with varying psychiatric comorbidity profiles to identify brief format efficacy limits, enabling precise risk stratification.
- **Process evaluation and delivery modality assessments** validating whether proposed mediators (acceptance, defusion) indeed drive improvements, comparing in-person model efficacy vs. mHealth adaptations for healthcare system sustainability.

Thus, the presented ACT-IM integration not only equips health professionals with replicable tools but also elevates methodological standards for chronic disease behavioral intervention development. This protocol should serve as a model bridging theoretical evidence and clinical practice fragmentation in T2DM patient care.
